# Correction: Microstructure and high frequency electromagnetic parameters of the soft/soft (CoFe_2_O_4_)_*x*_ : (Ni_0.4_Cu_0.2_Zn_0.4_Fe_2_O_4_)_*y*_ nanocomposites

**DOI:** 10.1039/d6ra90066f

**Published:** 2026-07-06

**Authors:** Alex V. Trukhanov, Munirah A. Almessiere, Abdulhadi Baykal, Yassine Slimani, Ekaterina L. Trukhanova, Daria I. Tishkevich, Svetlana V. Podgornaya, Egor Kaniukov, Sergei V. Trukhanov

**Affiliations:** a National University of Science and Technology MISiS 119049 Moscow Russia; b Scientific-Practical Materials Research Centre of NAS of Belarus 220072 Minsk Belarus truhanov86@mail.ru; c Department of Biophysics, Institute for Research and Medical Consultations (IRMC), Imam Abdulrahman Bin Faisal University P. O. Box 1982 Dammam 31441 Saudi Arabia; d Department of Physics, College of Science, Imam Abdulrahman Bin Faisal University P. O. Box 1982 Dammam 31441 Saudi Arabia; e Department of Nanomedicine Research, Institute for Research and Medical Consultations (IRMC), Imam Abdulrahman Bin Faisal University P. O. Box 1982 Dammam 31441 Saudi Arabia

## Abstract

Correction for ‘Microstructure and high frequency electromagnetic parameters of the soft/soft (CoFe_2_O_4_)_*x*_ : (Ni_0.4_Cu_0.2_Zn_0.4_Fe_2_O_4_)_*y*_ nanocomposites’ by Alex V. Trukhanov *et al.*, *RSC Adv.*, 2022, **12**, 34020–34027, https://doi.org/10.1039/D2RA06711K.

The authors regret that they did not disclose at the time of submission related work that was undergoing peer review at the same time but was published after this paper.^1^ This work should have been cited at the time of submission. Both papers are based on the same samples and therefore contain some of the same data in Fig. 1 and 3. However, this paper relates to electrodynamic properties of the samples whereas ref. 1 below relates to magnetic properties of the samples.

The Royal Society of Chemistry apologises for these errors and any consequent inconvenience to authors and readers.
